# Inter-Individual and Inter-Strain Variations in Zebrafish Locomotor Ontogeny

**DOI:** 10.1371/journal.pone.0070172

**Published:** 2013-08-09

**Authors:** Merlin Lange, Frederic Neuzeret, Benoit Fabreges, Cynthia Froc, Sebastien Bedu, Laure Bally-Cuif, William H. J. Norton

**Affiliations:** 1 Zebrafish Neurogenetics, Neurobiologie et Développement, Insitut de Neurobiologie Albert Fessard, Centre National de la Recherche Scientifique, Gif-sur-Yvette, Essonne, France; 2 ViewPoint Life Sciences, Lissieu, Rhône, France; 3 Département de Mathématiques, Université Paris-Sud 11, Orsay, Essonne, France; 4 Department of Biology, University of Leicester, Leicester, Leicestershire, United Kingdom; Institut Curie, France

## Abstract

Zebrafish exhibit remarkable alterations in behaviour and morphology as they develop from early larval stages to mature adults. In this study we compare the locomotion parameters of six common zebrafish strains from two different laboratories to determine the stability and repeatability of these behaviours. Our results demonstrate large variability in locomotion and fast swim events between strains and between laboratories across time. These data highlight the necessity for careful, strain-specific controls when analysing locomotor phenotypes and open up the possibility of standardising the quantification of zebrafish behaviour at multiple life stages.

## Introduction

The study of behavioural ontogeny, the development of behaviour throughout life, is necessary in order to fully understand an animal's behavioural repertoire. The ethologist Niko Tinbergen proposed that ontogeny is one of the four main questions which can be used to study behaviour [1]. Ontogeny, as defined by Tinbergen, is a developmental change in the machinery (such as neural circuits or hormone systems) that underlies behaviour rather than alterations in behaviour itself [1]. Both genetic and environmental factors interact to modify the expression of behaviour. However, under laboratory conditions where environmental influences are standardised, the influence of genetic background on behaviour over time can be studied.

The zebrafish is a popular model for developmental biology which has also been used to investigate the genetics of behaviour [2], [3]. Larval zebrafish are small, translucent and easy to characterise anatomically. They are also genetically tractable and can be used for live recordings of cell-type-specific fluorescent reporters, laser ablations, electrophysiology and optogenetic manipulation [4]–[9]. The establishment of the *casper* mutant line, which remains transparent throughout its life [10] raises the possibility of extending these techniques to adult fish. Since zebrafish are fertilised outside of their mother their development and behaviour can be systematically studied at different life stages making them ideal for longitudinal studies. However, despite numerous reports of behaviour at either larval (6 or 7 day-old) or adult (3 months or older) stages, there have been few comparisons of behaviour during juvenile development (but see [11], [12]). Furthermore, there appear to be no studies that measure the stability of behaviour in a single group of fish throughout their life.

Zebrafish are also increasingly used as a translational model for human disease. Many of these models are based upon behavioural analysis of larval fish and include measurements of locomotion. For example, hyperactivity (increased locomotion) has been used as an endophenotype to study the function of attention-deficit/hyperactivity disorder (ADHD)-linked genes in zebrafish [13]. Exposure to Parkinson's disease-linked toxins or knock-down of Parkinson's disease susceptibility genes can cause a reduction of swimming at larval stages [14]. Reduced locomotion following touch has also been used to assess the function of *SHANK3*, a gene which is connected to autism spectrum disorder in humans [15]. Finally, prepulse inhibition, the habituation of a startle response by a preceding non-startling stimulus has been used as an endophenotype for schizophrenia in zebrafish [16]. It is therefore essential to define the baseline parameters for locomotor behaviour. This information will facilitate high-throughput experiments as well as the standardisation of the assessment protocols to measure locomotion in different laboratories.

In this study we have measured changes to locomotion in six different zebrafish strains across their lifespan to document the variability of this behaviour over time. We have also examined fish originating from a different laboratory to examine the repeatability of our behavioural measures. We have developed software to automatically quantify the distance travelled, speed and resting time for 6-day, 1-month- and 3-month-old zebrafish. In parallel, we have created a macro to assess fast swim events in these animals. The methods that we present here provide a robust and precise way to study zebrafish behavioural variability in a high-throughput manner. Together, these data provide us with an insight into the stability of locomotion over time, as well as some of the genetic and environmental factors that might influence it.

## Materials and Methods

### Ethics statement

All protocols have been approved by the local competent authority (authorisation A91-577 of the Prefecture de l'Essonne, France).

### Animal strains, care and maintenance

Adult zebrafish were maintained using standard fish-keeping protocols. Locomotion was recorded in an isolated room with standardised lighting and heating to minimise environmental variation and interference from background noise during testing. All experiments were performed on embryos or adults of six different zebrafish strains. Five of these strains were inbred for at least 6 generations (AB), or 3 generations (*casper*, Ekkwill (EK), Tuebingen (TU) and Wild India Karyotype (WIK)) from stocks in our Gif-sur-Yvette facility. *casper* is a transparent double mutant line formed by crossing *nacre*/*mitfa* with *roy* [10]. In order to discriminate between genetic inbreeding and the influence of environment, we tested AB fish originating from Strasbourg, France (gift from O. Pourquié and M. Rebagliati, IGBMC). “ABstrg” adults were acclimatised to our facility for 6 months before being used to produce embryos for these experiments. Each fish was mated at least twice to check stability of the results that we observed between experiments. After collection, all eggs were kept in embryo medium [17] in a 28.5°C incubator with a 14-hour day/night light cycle (lights on at 8 am and off at 10 pm) in groups of 40 fish per Petri dish. On day 6, larval locomotion was tested between 1 pm and 4 pm with an ambient room temperature of approximately 27°C. Directly after the experiment, embryos were transferred to small aquaria (AquaBox 3, Schwarz GmbH, Germany, dimensions 24.5 cm×15 cm×13.5 cm) at a concentration of 40 embryos per box. Dead embryos were removed on day 20 post fertilisation and the density of fish in the aquarium was homogenised to maintain groups of approximately 20 fry. At 30 days post fertilisation, fish were transferred to the behaviour testing room and allowed to habituate to their new environment. Tanks were connected to a continuous water flow that changed about 10% of the total filtered facility water each day. The temperature and light cycle were identical to the main holding facility. Fish were fed three times a day. Fish younger than 20 days old were fed with a mixture of NovoTom artemia (JBL GmbH, Germany) and live artemia nauplii. Adults were fed a morning and evening feed with dry flakes (TetraMin, Tetra GmbH, Germany) and a lunchtime feed with artemia nauplii (INVE aquaculture, Belgium). Animals were placed back in the main fish facility between behavioural recordings at one month and three months to maintain fish in optimal environmental conditions. Therefore, all larvae, juveniles and adults tested in this study were raised and recorded under standardised conditions. We chose AB as our reference strain for all experiments since it is commonly used for behavioural analysis and has a well-documented life history (http://zfin.org/action/genotype/genotype-detail?zdbID=ZDB-GENO-960809-7).

### Experimental apparatus and design

#### Larval stage (6 days post fertilisation)

Locomotor activity was examined at 6 days post fertilisation (dpf) by recording larval swimming during a 1-hour period using ZebraLab software (ViewPoint Life Sciences, France; tracking parameters are described below). Larvae were placed into separate wells of a 24-well plate (BD Falcon GmBH, Germany) inside a ZebraBox (ViewPoint Life Sciences, France; Fig. S1). Fry were gently pipetted into the plate 1 hour before the experiment started. Multi-well plates were placed into the ZebraBox and larvae were allowed to habituate for 10 minutes before recording began. A schematic representation of the behavioural setup used at each life stage is provided in figure S1.

#### Juvenile stage (1 month)

At one month the fish were individually placed in a small transparent tank (9.5 cm×6 cm×4.5 cm) filled with 100 ml of filtered facility water. Following a 24-hour acclimation period fish were imaged for a 1-hour period between 1 pm and 4 pm. Boxes were placed in a ViewPoint ZebraCube (a cubicle in which the day/night light cycle and environment can be controlled; ViewPoint Life Sciences, France, Fig. S1) which allows up to 40 individual 1 month-old zebrafish to be monitored at the same time. We placed separations between the boxes so that juvenile fish could not see each other.

#### Adult stage (3 months)

The last experiment was performed at 3 months, a stage where zebrafish are sexually mature and so are considered to be adult. We recorded the number of male and female fish at the end of the experiment, and did not see large variation in sex ratio between strains: AB, 42% female, 58% male; ABstrg, 53% female, 47% male; *casper*, 60% female, 40% male; EK, 42% female, 58% male; TU, 50% female, 50% male; WIK 40% female, 60% male. Furthermore, when analysing results, we did not see an obvious correlation between sex ratio and distance swum. For instance, AB (42% female, 58% male) and *casper* (60% female, 40% male) showed different sex ratios, but swam a similar distance at 3 months. Fish were permitted to habituate for 24 hours before the experiment and were recorded during a 1-hour session between 1 pm and 4 pm. Individual fish were gently placed in an AquaBox 3 (Schwarz GmBH, Germany) filled with 2.5 litres of filtered facility water. We constructed a large chamber that allowed 24 adult fish to be recorded at the same time (Fig. S1). Tanks were placed on an infrared floor and the camera fixed 190 cm from the fish.

### Quantification of behaviour

#### ZebraLab parameters

The following parameters were used in the ZebraLab programme: transparent background mode with a threshold of 11 at 6 dpf, 1 month, and 3 months. Fish were illuminated with both infrared light and white light (100 lux inside the ZebraBox (for 6 dpf larvae), 69 lux in the ZebraCube (for 1 month-old juveniles) and 75 lux in the adult setup). The same camera was used to record behaviour at both 1 month and 3 months. The camera was calibrated to detect infrared light and was set to 25 frames per second.

#### Locomotion

Locomotion parameters were measured using an automated live video tracking system (ZebraLab, ViewPoint Life Sciences, France). Using a high-speed infrared camera the fry were tracked for 1 hour. The integration period (the time intervals used to measure distance swum in each experiment) was 1 minute for 6 dpf larvae, and 5 minutes for 1 month and 3 month-old fish. These data were then exported into FastData Monitor, a software package developed in collaboration with ViewPoint Life Sciences. Several parameters were extracted to look at locomotion in greater detail including distance swum, swimming time and mean speed during one hour. The mean speed of swimming was calculated by dividing the total distance swum by the amount of time spent swimming (not the total length of the recording period); thus frames in which no movement was detected were excluded from this analysis.

#### Fast swim events

Fast swim events were quantified by counting the number of events where the fish swim more than 5 mm in less than 12 seconds [13]. The ZebraLab software was used to record locomotion during 5 minutes with an integration period of 3 seconds. The tracking parameters used were the same as those described above. Data were exported into Excel (Microsoft) and the peak parameters automatically sorted using a self-designed macro (details available upon request).

#### Surface and speed

The relative surface area of the fish was measured using the “size” extension of the ZebraLab software (ViewPoint), which allowed the surface area of the fish to be quantified in pixels. Pixel threshold was set at 117 in the transparent background mode for 1 month-old and 3 month-old fish. At 1 month, 1 pixel corresponded to 2.23 mm^2^, and at 3 months to 3.61 mm^2^. The locomotion tracking parameters used in this experiment were identical to those described above. We did not include 6 dpf larvae in this test because the difference between individuals in relative pixel size was not significant at this stage. Surface data were exported and analysed with SigmaPlot 2.0 (SystaSoftware, Inc, Chicago, USA).

### Behavioural analysis using FastData Monitor software

One of the challenges of high-throughput behavioural analysis is to sort and extract data in a simple way. We have developed software to extract behavioural information from raw data in collaboration with ViewPoint Life Sciences (France). The FastData Monitor software is able to filter and sort the result files and can be used for routine calculations such as sums, means and standard deviations without the need to learn a programming language. Finally, the user is able to choose between different types of charts including column, pie, bar or stacked column charts in order to visualise results. These settings can be saved in a layout file and reused thus standardising the processing and reporting of data. The behavioural parameters presented in this study have been extracted from films of zebrafish locomotion using FastData Monitor. In this experiment only one area of interest corresponding to an individual aquarium was defined. However, multiple areas of interest can be defined within ZebraLab allowing the preference of a fish for different areas within an aquarium to be measured [18]. The resting time corresponds to the amount of time spent inactive during data acquisition and distance, speed and resting time can be calculated for both individual animals and groups of fish.

To develop the FastData Monitor software Microsoft Visual Studio (Microsoft, USA) was used in conjunction with the. NET framework (Microsoft, USA) on Windows 7. It is able to process data contained in an Excel sheet. The user interface also allows novel customised formulae to be defined based on columns and rows of data. The software then produces layouts that contain the sorting parameters and formulae to be applied to the input data as well as the chart representations that will be generated at the end of the experiment. Layouts can be reused to reproduce the same operations on different data. FastData Monitor can standardise data manipulation and chart production across laboratories thereby improving the reliability and repeatability of behavioural experiments. A basic version of the FastData Monitor software is available for use via the following link: http://fastdatamonitor.vplsi.com/download/BasicFastDataMonitorSetup.msi. The software requires that data are uploaded as an Excel file in a specific format. More details regarding this issue will be provided upon request.

### Statistical analysis

We have used AB as a reference strain throughout this study to simplify the presentation of results. However, we have also indicated significant differences between other strains where possible. All error bars denote SEM. Statistical significance was depicted as follows: NS (non-significant), *p*>0.05; *p<0.05, **p<0.01, ***p<0.001. In all cases the number of animals tested is denoted by n. Strains of the same stage were compared using Student's t-tests on independent samples assuming equal variance and performed using Excel (Microsoft, USA). Analysis of variance (ANOVA) and a Bonferroni correction were performed on the surface data using Aabel 3 (Gigawiz, USA). To compare the differences between activity thresholds the arcsine transformation was used to correct the skew that might be generated by using percentages as raw data for statistical analyses [19]. For the surface and speed a Pearson product-moment correlation coefficient followed by Student's t-distribution was performed using Aabel 3 (Gigawiz, USA).

## Results

In order to compare genetic and environmental influences on behaviour across time we measured locomotion in six strains of zebrafish at 6 days, 1 month and 3 months post fertilisation. We compared the behaviour of AB, Ekkwill (EK), Tuebingen (TU) and Wild India Karyotype (WIK) fish that were raised in the Institute of Neurobiology Albert Fessard, Gif-sur-Yvette, France. We also analysed *casper*, a transparent mutant line which is used for optogenetics and live imaging [10]. Finally, in order to compare variations in the behaviour of zebrafish maintained in different labs, we quantified the locomotion of progeny collected from AB fish that were born and raised in the Institute of Genetics and Molecular and Cellular Biology (IGBMC), Strasbourg, France and transferred to Gif-sur-Yvette as adults. We collected embryos for each of these strains and made repeated measurements of locomotion and fast swim events as the fish grew to maturity.

### Locomotion at 6 days post fertilisation

**Figure 1:**
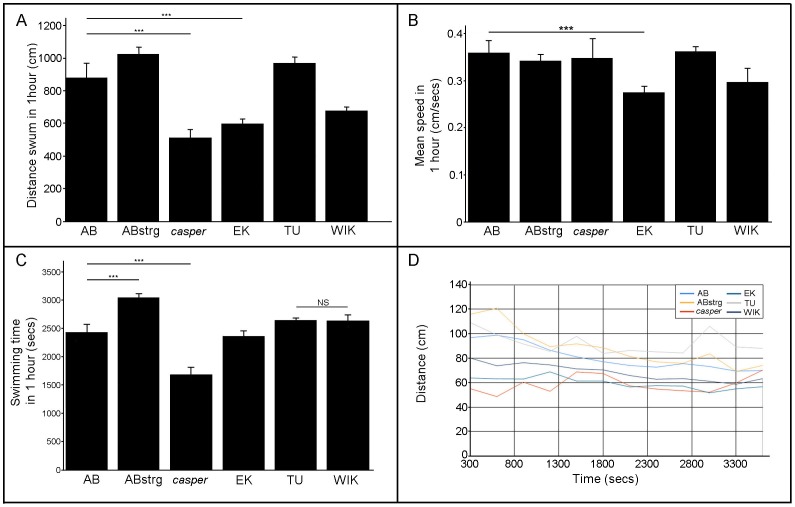
Locomotory behaviour of six zebrafish strains at 6 days post fertilisation. A) Mean distance swum in a 60-minute time interval by 6 day-old AB, ABstrg, *casper*, EK, TU and WIK larvae. *casper*, EK and WIK larvae swam significantly less than AB, ABstrg or TU. For the statistics refer to table 2. AB n = 47; ABstr n = 94; *casper* n = 47; EK n = 90; TU n = 47 and WIK n = 93. B) Mean swimming speed of 6 day-old AB, ABstrg, *casper*, EK, TU and WIK larvae during a 60-minute experiment. EK and WIK larvae swam significantly slower than the other fish strains. For the statistics refer to table 2. AB n = 47; ABstr n = 94; *casper* n = 47; EK n = 90; TU n = 47 and WIK n = 93. C) Mean time spent swimming during a 60-minute experiment for 6 day-old AB, ABstrg, *casper*, EK, TU and WIK larvae. ABstrg swam significantly more than all other fish strains. *casper* swam significantly less than all other fish strains. EK swam significantly less time than ABstrg, WIK and TU, and significantly more than *casper*. For the statistics refer to table 2. AB n = 47; ABstr n = 94; *casper* n = 47; EK n = 90; TU n = 47 and WIK n = 93. D) The distance swum every 5 minutes for each strain as function of time. Values are plotted every 300 seconds in a 60-minute experiment for 6 day-old AB, ABstrg, *casper*, EK, TU and WIK larvae. AB, ABstrg and EK fish show smoother locomotion curves than *casper*, TU and WIK. For the statistics refer to table 1. AB n = 47; ABstr n = 94; *casper* n = 47; EK n = 90; TU n = 47 and WIK n = 93.

We first compared the swimming behaviour of larval fish at 6 days post fertilisation (dpf). At this stage of development, larvae have a paired fin fold, relatively underdeveloped pectoral fins and a prominent yolk sac. We measured locomotion during a 60-minute experiment and used ZebraLab to quantify behaviour. We first analysed the total distance swum. ABstrg, TU and WIK larvae swam a similar distance to AB, whereas *casper* and EK swam a significantly shorter distance (Fig. 1A). We next looked at the average swimming speed during a one-hour recording. Interestingly, the speed of swimming appeared to show no correlation to the distance swum; AB, ABstrg, *casper* and TU swam a similar speed whereas EK and WIK swam significantly slower (Fig. 1B). The total distance swum by fish could also be related to the amount of time spent swimming. In a one-hour time period, AB fish spent on average 2400 seconds swimming (equivalent to 40 minutes) with no significant differences for EK, TU or WIK (Fig. 1C). ABstrg spent significantly more, and *casper* significantly less time swimming than AB (Fig. 1C). To determine whether these phenotypes were the result of stable differences over time we plotted the distance swum every 5 minutes during a one-hour experiment (Fig. 1D). Groups of AB, ABstrg and TU fish swam consistently further than *casper*, EK and WIK during this experiment (Fig. 1D; for statistics, refer to table 1). In summary, at 6 dpf, fish of different strains showed significant variation in the amount and pattern of larval locomotion (for statistics, refer to table 2).

**Table 1:** Summary of the two-way ANOVA followed by Bonferroni-Dunn Test for the sum of the mean total distance swum for each strain as function of time described in Fig. 1D, Fig. 2D and Fig. 3D.

	6 dpf		1 Month		3 Months	
	Two-way ANOVA		(independent samples)			
	*df*	*Mean Square*	*df*	*Mean Square*	*df*	*Mean Square*
*Between groups*	5	2583.97	5	7.00E+08	5	588792
*Within groups (error)*	78	6.06E+07	78	6.06E+07	66	16365.3
			**Bonferroni**	**Dunn Test**		
			*α = 0.05*			
			*Adjusted α*	* = 0.0033*		
*Groups*	*p*	*Significant*	*p*	*Significant*	*p*	*Significant*
AB vs ABstrg	0.419	No	<0.001	Yes	<0.001	Yes
AB vs *casper*	<0.001	Yes	<0.5	No	<0.5	No
AB vs EK	<0.001	Yes	<0.5	No	<0.001	Yes
AB vs TU	0.006	No	<0.5	No	<0.001	Yes
AB vs WIK	<0.001	Yes	<0.5	No	<0.001	Yes
ABstrg vs *casper*	<0.001	Yes	<0.001	Yes	<0.001	Yes
ABstrg vs EK	<0.001	Yes	<0.001	Yes	<0.001	Yes
ABstrg vs TU	0.046	No	<0.001	Yes	0.006	No
ABstrg vs WIK	<0.001	Yes	<0.001	Yes	<0.5	No
*casper* vs EK	<0.5	No	<0.5	No	<0.001	Yes
*casper* vs TU	<0.001	Yes	<0.5	No	<0.001	Yes
*casper* vs WIK	0.014	No	<0.5	No	<0.001	Yes
EK vs TU	<0.001	Yes	<0.5	No	<0.01	No
EK vs WIK	0.031	No	<0.5	No	<0.001	Yes
TU vs WIK	0.003	Yes	<0.5	No	0.025	No

**Table 2:** Summary of the different P-values following Student's t-test for the locomotion (distance, speed, time) and threshold experiment described in Fig. 1 A,B,C,E, Fig. 2 A,B,C,E, Fig. 3 A,B,C,E at 6 dpf, 1 month and 3 months.

								Locomotion		Statistics									
				Distance						Speed						Time			
		AB	ABstrg	*casper*	EK	TU	WIK	AB	ABstrg	*casper*	EK	TU	WIK	AB	ABstrg	*casper*	EK	TU	WIK
6 dpf	AB	-	NS	**	**	NS	NS	-	NS	NS	**	NS	NS	-	***	***	NS	NS	NS
6 dpf	ABstrg	NS	-	***	***	NS	***	NS	-	NS	*	NS	NS	***	-	***	***	***	*
6 dpf	*casper*	**	***	-	NS	***	***	NS	NS	-	NS	NS	NS	***	***	-	***	***	***
6 dpf	EK	**	***	NS	-	***	*	**	*	NS	-	***	NS	NS	***	***	-	**	*
6 dpf	TU	NS	NS	***	***	-	***	NS	NS	NS	***	-	*	NS	***	***	**	-	NS
6 dpf	WIK	NS	***	***	**	***	-	NS	NS	NS	NS	*	-	NS	*	***	*	NS	-
1month	AB	-	*	NS	NS	NS	NS	-	***	NS	NS	NS	NS	-	***	NS	**	***	***
1month	ABstrg	*	-	NS	***	***	***	***	-	**	***	**	**	***	-	***	NS	NS	NS
1month	*casper*	NS	NS	-	***	***	**	NS	**	-	NS	NS	NS	NS	***	-	**	***	**
1month	EK	NS	***	***	-	NS	NS	NS	***	NS	-	NS	NS	**	NS	**	-	NS	NS
1month	TU	NS	***	***	NS	-	NS	NS	**	NS	NS	-	NS	***	NS	***	NS	-	NS
1month	WIK	NS	***	**	NS	NS	-	NS	**	NS	NS	NS	-	***	NS	***	NS	NS	-
3month	AB	-	***	NS	NS	***	***	_	NS	NS	NS	NS		-	***	***	**	***	***
3month	ABstrg	***	-	***	***	NS	NS	NS	-	***	*	NS	**	***	-	NS	NS	NS	**
3month	*casper*	NS	***	-	NS	***	***	NS	***	-	NS	NS	NS	***	NS	-	NS	NS	**
3month	EK	NS	***	NS	-	*	***	NS	*	NS	-	NS	NS	**	NS	NS	-	NS	***
3month	TU	***	NS	***	*	-	NS	NS	NS	NS	NS	-	NS	***	NS	NS	NS	-	NS
3month	WIK	***	NS	***	***	NS	-	NS	**	NS	NS	NS	-	***	**	**	***	NS	-

NS (non-significant), *p*>0.05;

* *p*<0.05;

** *p*<0.01;

*** *p*<0.001.

### Locomotion at 1 month post fertilisation

**Figure 2:**
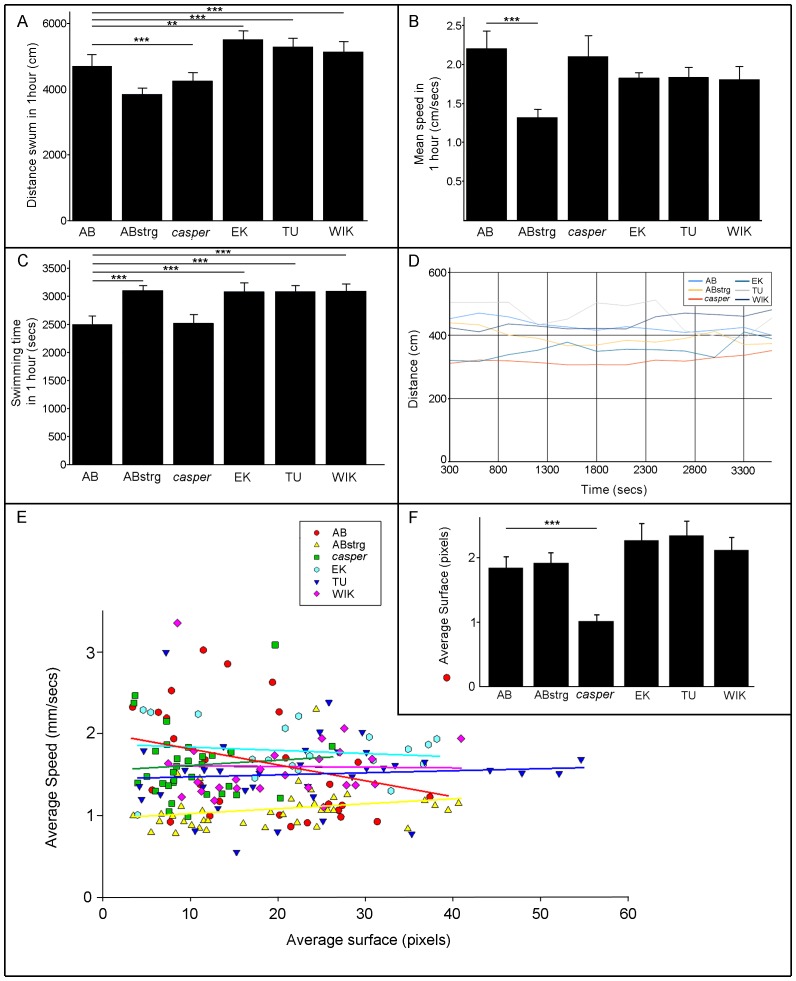
Locomotory behaviour of six zebrafish strains at one month post fertilisation. A) Mean distance swum in a 60-minute time interval by 1 month-old AB, ABstrg, *casper*, EK, TU and WIK juveniles. ABstrg swam significantly less than all other strains apart from *casper*. *casper* swam significantly less than EK, TU and WIK. For the statistics refer to table 2. AB n = 39; ABstrg n = 39; *casper* n = 37; EK n = 19; TU n = 39, WIK n = 27. B) Mean swimming speed of 1 month-old AB, ABstrg, *casper*, EK, TU and WIK juveniles during a 60-minute experiment. ABstrg swam significantly slower than the other fish strains. For the statistics refer to table 2. AB n = 39; ABstrg n = 39; *casper* n = 37; EK n = 19; TU n = 39, WIK n = 27. C) Mean time spent swimming during a 60-minute experiment for 1 month-old AB, ABstrg, *casper*, EK, TU and WIK juveniles. ABstrg, EK, TU and WIK swam for significantly more time than AB and *casper*. For the statistics refer to table 2. AB n = 39; ABstrg n = 39; *casper* n = 37; EK n = 19; TU n = 39, WIK n = 27. D) The distance swum every 5 minutes for each strain as function of time. Values are plotted every 300 seconds in a 60-minute experiment for 1 month-old AB, ABstrg, *casper*, EK, TU and WIK juveniles. AB, ABstrg, *casper*, EK and WIK fish show smoother locomotion curves than TU. For the statistics refer to table 1. AB n = 39; ABstrg n = 39; *casper* n = 37; EK n = 19; TU n = 39, WIK n = 27. E) Graph showing the correlation between fish size (measured by surface area) and swimming speed during a 60-minute experiment for one month old AB, ABstrg, *casper*, EK, TU and WIK juveniles. There is no correlation between size of fish and swimming speed for any strain analysed. We calculated the Pearson product-moment coefficient for each strain (*r*) with speed as independent variable and surface as dependant variable. AB *r* = 0.23 NS; ABstrg *r* = −0.28 NS; *casper r* = 0.07 NS; EK *r* = −0.12 NS; TU *r* = 0.17 NS; WIK *r* = 0.3 *p = 0.05. AB n = 29; ABstrg n = 39; *casper* n = 32; EK n = 19; TU n = 38, WIK n = 26. F) Relative size of 1 month-old juvenile fish, calculated by measuring the average number of pixels making up their surface area. *casper* mutant fish have a significantly smaller surface area than AB, ABstrg, EK, TU and WIK. Student's t-test: AB vs ABstrg NS; AB vs *casper* ***p<0.001; AB vs EK NS; AB vs TU NS; AB vs Wik *p<0.05; ABstrg vs *casper* ***p<0.001; ABstrg vs EK NS; ABstrg vs TU NS; ABstrg vs WIK NS; *casper* vs EK ***p<0.001; *casper* vs TU ***p<0.001; *casper* vs WIK ***p<0.001; EK vs TU NS; EK vs WIK NS; TU vs WIK NS.

After one month of development juvenile fish have more pronounced pectoral, caudal, dorsal and pelvic fins. The median fin fold is reduced in size and the yolk has been absorbed to form a more streamlined body [20]. We measured the total distance swum by all fish strains during a one-hour time period. At this stage, ABstrg and *casper* swam a significantly shorter distance than AB, whereas EK, TU and WIK swam significantly further (Fig. 2A). When comparing the average speed of swimming for the different groups of fish, ABstrg juveniles swam significantly slower than all other strains (Fig. 2B). In contrast to this however, the amount of time swimming showed a different pattern. AB fish spent around 2505 seconds (approximately 42 minutes) swimming in a one-hour time period (Fig. 2C). *casper* juveniles swam for a similar amount of time as AB, whereas ABstrg, EK, TU and WIK all swam significantly more (Fig. 2C). When looking at global activity levels (calculated by plotting the total distance swum every 5 minutes during a one hour experiment), AB, EK and TU juveniles swam consistently further than ABstrg, *casper* and WIK (Fig. 2D; for statistics, refer to table 1). We also compared the surface area of these animals (which is an indirect measure of their overall size; 1 pixel corresponds to 2.23 mm^2^) to the average swimming speed. Lines of best fit applied to these data showed no correlation between the size of a fish and speed at which it swam (Fig. 2E). However, this analysis did reveal that *casper* juveniles had a much smaller surface area than fish of other strains at this age (Fig. 2F). In summary, at one month, ABstrg juveniles swam a shorter distance and slower than the other fish strains analysed suggesting an influence of the parental environment or genotype on these aspects of locomotion. Conversely, AB, *casper*, EK, TU and WIK showed fairly homogenous distance, speed and time of swimming (for statistics, refer to table 2). There also appeared to be little, if any, correlation between the size of an animal and the speed of its swimming.

### Locomotion at 3 months post fertilisation

**Figure 3:**
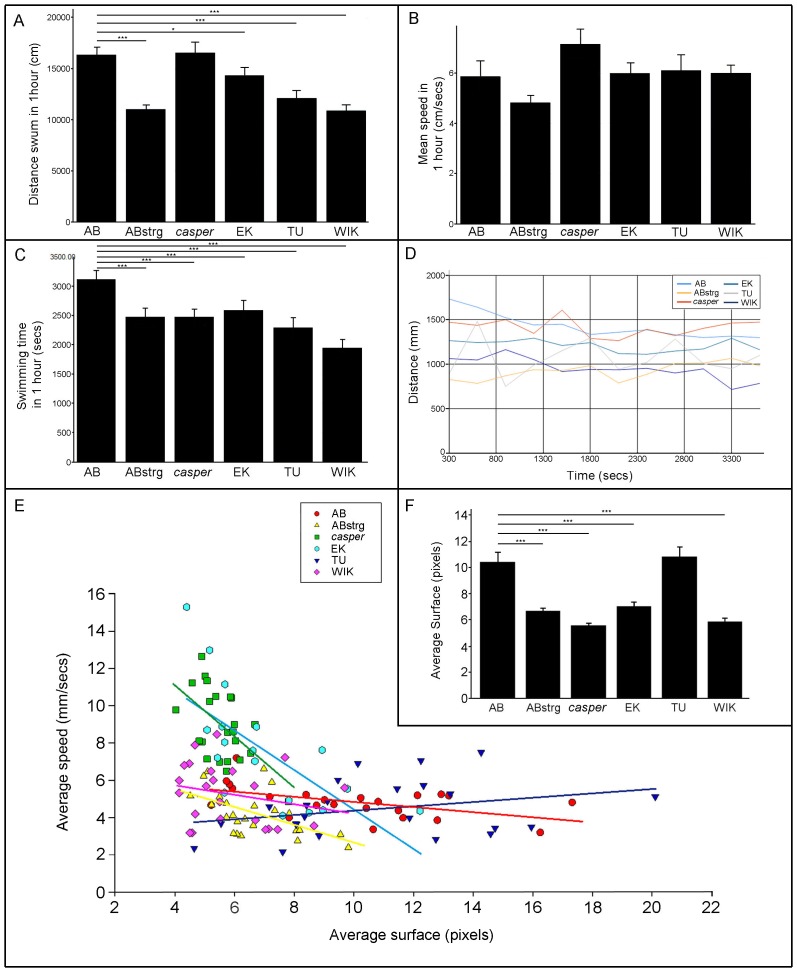
Locomotion profiles of six zebrafish strains at three months post fertilisation. A) Mean distance swum in a 60-minute time interval by 3 month-old AB, ABstrg, *casper*, EK, TU and WIK adults. ABstrg, TU and WIK swam significantly less than AB, *casper* and EK. For the statistics refer to table 2. AB n = 19; ABstrg n = 22; *casper* n = 21; EK n = 18; TU n = 23, WIK n = 23. B) Mean swimming speed of 3 month-old AB, ABstrg, *casper*, EK, TU and WIK adults during a 60-minute experiment. ABstrg swam significantly slower than the other fish strains. For the statistics refer to table 2. AB n = 19; ABstrg n = 22; *casper* n = 21; EK n = 18; TU n = 23, WIK n = 23. C) Mean time spent swimming during a 60-minute experiment for 3 month-old AB, ABstrg, *casper*, EK, TU and WIK adults. ABstrg, *casper*, EK, TU and WIK swam significantly less time than AB. For the statistics refer to table 2. AB n = 19; ABstrg n = 22; *casper* n = 21; EK n = 18; TU n = 23, WIK n = 23. D) The distance swum every 5 minutes for each strain as function of time. Values are plotted every 300 seconds in a 60-minute experiment for 3 month-old AB, ABstrg, *casper*, EK, TU and WIK adults. AB, ABstrg, *casper* and WIK fish show smoother locomotion curves than EK and TU. For the statistics refer to table 1. AB n = 19; ABstrg n = 22; *casper* n = 21; EK n = 18; TU n = 23, WIK n = 23. E) Graph showing the correlation between fish size (measured by surface area) and swimming speed during a 60-minute experiment for 3 month-old AB, ABstrg, *casper*, EK and TU adults. At this age there appears to be a correlation between size of fish and swimming speed for ABstrg, *casper* and WIK. Similar to our analysis at 1 month, we determined the Pearson product-moment coefficient for each strain (*r*) with speed as the independent variable and surface as dependant variable to investigate surface and speed. AB *r* = −0.57 **p<0.025: NS; ABstrg *r* = −0.54 **p<0.025; *casper r* = −0.39 *p<0.05; EK *r* = −0.68 ***p<0.001; TU *r* = 0.28 NS; WIK *r* = −0.23 NS. AB n = 24; ABstrg n = 24; *casper* n = 24; EK n = 18; TU n = 23, WIK n = 23. F) Relative size of 3 month-old adult fish calculated by measuring the average number of pixels making up their surface area. ABstrg, *casper*, EK and WIK adults have a significantly smaller surface area than AB and TU. Student's t-test: AB vs ABstrg ***p<0.001; AB vs *casper* ***p<0.001; AB vs EK ***p<0.001; AB vs TU NS; AB vs Wik ***p<0.001; ABstrg vs *casper* ***p<0.001; ABstrg vs EK NS; ABstrg vs TU ***p<0.001; ABstrg vs WIK NS; *casper* vs EK **p<0.025; *casper* vs TU ***p<0.001; *casper* vs WIK NS; EK vs TU ***p<0.001; EK vs WIK P *p<0.05; TU vs WIK ***p<0.001.

After 3 months of development the zebrafish strains that we analysed were able to reproduce and so were deemed to be sexually mature. These fish had fully developed adult fins (including pectoral, caudal, pelvic and anal fins) and a streamlined body. We first measured the total distance swum by these adult fish during one hour. At this stage of life ABstrg, TU and WIK swam a significantly shorter distance than AB, *casper* or EK without being significantly different to each other (Fig. 3A). During the same experiment there was no significant difference in the speed of swimming between all the strains analysed (Fig. 3B). In contrast to this, ABstrg, *casper*, EK, TU and WIK swam for a shorter period of time than AB (Fig. 3C). However, AB and *casper* adults swam consistently further than all other strains (Fig. 3D). The amount of variation in activity levels appeared to be similar in 3-month old adults compared to 1-month old juveniles. At 3 months we found evidence of a correlation between a fish's size and its average swimming speed. AB, ABstrg, *casper* and EK show a strong negative correlation between size and speed with larger animals swimming slower (Fig. 3E). There was a weaker correlation in the same direction for TU and no correlation for WIK (Fig. 3E). Interestingly, at this age the size of fish (judged by the number of pixels making up their surface area; 1 pixel corresponds to 3.61 mm^2^) was much more variable than at 1 month. ABstrg, *casper*, EK and WIK adult were much smaller than AB or TU (Fig. 3F) despite being maintained in similar conditions in the aquarium. In summary, at 3 months of age there was a significant variation in the distance swum by each fish strain. However, the speed and amount of time spent swimming was much more homogenous (for statistics, refer to table 2). Even though the fish differed in size between strains, a correlation between surface area and the speed of swimming was apparent with larger animals generally swimming less. The feeding regime and environmental conditions were the same for all fish strains suggesting that genetic differences between these fish strains might account for the variation in surface area. Since large fish have a higher spawning frequency and larval hatching rate than small fish, the variation in locomotion linked to size we observe might provide an indirect readout of global fitness [21].

### The ontogeny of locomotion

**Figure 4:**
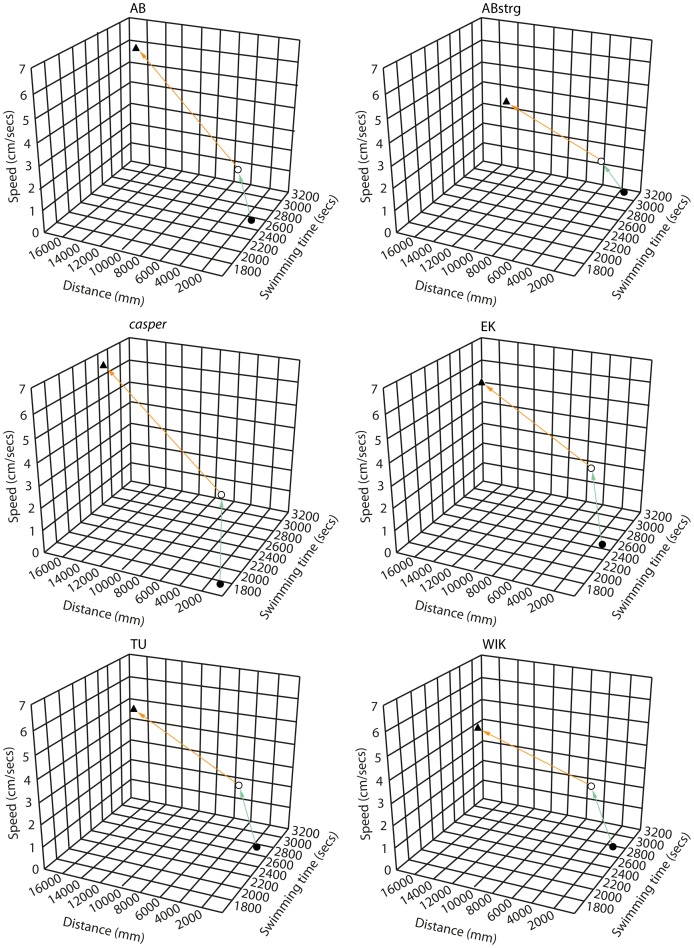
The ontogeny of zebrafish locomotion. Graph showing a summary of behavioural ontogeny in 6 zebrafish strains. The black circles represent mean data points for 6 day-old larvae, clear circles for 1 month-old- and black triangles for 3 month-old fish. The arrows indicate the increase in distance, speed and time throughout the experiment.

By repeatedly measuring the same group of animals we were able to determine alterations to behaviour as fish develop to adulthood. Several trends became apparent when analysing these data (Fig. 4). Firstly, both the mean distance and the speed of swimming significantly increased over time. AB fish swam 881 cm at 0.36 cm/sec at 6pdf, 4719 cm at 2.21 cm/sec at 1 month and 16370 cm at 5.87 cm/sec at 3 months (Fig. 1A,B,C; 2A,B,C; 3A,B,C: AB speed at each stage was compared by ANOVA followed by Bonferroni-Dunn post-hoc analysis. AB 6 dpf vs AB 1 month ***p<0.001; AB 1 month vs AB 3 months ***p<0.001. AB distance at each stage was compared by ANOVA followed by Bonferroni-Dunn post-hoc analysis. AB 6 dpf vs AB 1 month ***p<0.001; AB 1 month vs AB 3 months ***p<0.001). In contrast, the amount of time spent swimming remains relatively constant with AB swimming for 2435 seconds at 6 dpf and 2506 seconds at 1 month with a significant increase to 3116 seconds at 3 months (AB distance at each stage was compared by ANOVA followed by Bonferroni-Dunn post-hoc analysis. AB 6 dpf vs AB 1 month NS; AB 1 month vs AB 3 months ***p<0.001).

### Fast swim events throughout fish maturation

**Figure 5:**
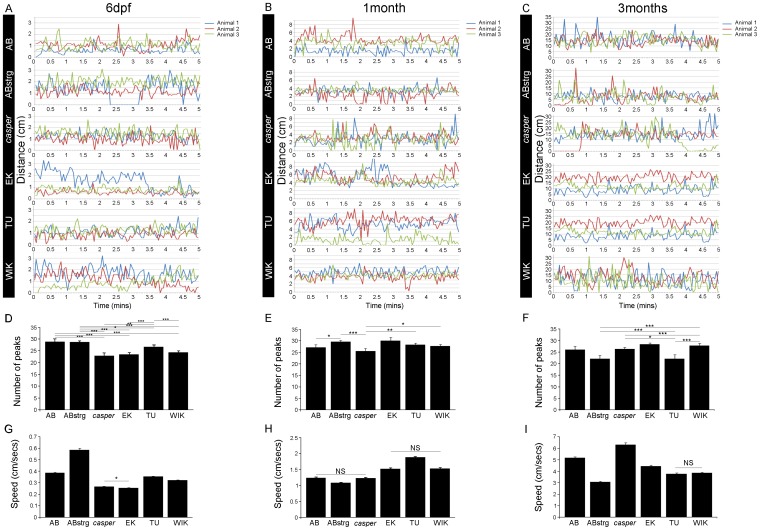
Fast swim event curves for 6 zebrafish strains at different life stages. A,B,C) Representative curves showing fast swim events in three individual fish at 6 dpf (A), 1 month (B) and 3 months (C). Strains are indicated on black bars to the left. D) Number of peaks of fast swimming during a 5-minute experiment. Peaks were defined as acceleration events when the larvae travelled more than 5 mm in less than 12 seconds. At 6 dpf, *casper*, EK, TU and WIK show fewer peaks of fast swimming than AB and ABstrg. For the statistics refer to table 3. E) Number of peaks of fast swimming during a 5-minute experiment. Peaks were defined as acceleration events when the juveniles travelled more than 5 mm in less than 12 seconds. At 1 month ABstrg, EK, TU and WIK show more peaks of fast swimming than AB and *casper*. For the statistics refer to table 3. F) Number of peaks of fast swimming during a 5-minute experiment. Peaks were defined as acceleration events when the fish travelled more than 5 mm in less than 12 seconds. At 3 months ABstrg and TU showed significantly fewer peaks of fast swimming than AB, *casper*, EK and WIK. There were, however, differences between the other strains (see results). For the statistics refer to table 3. G) Mean distance swum in a 5-minute time interval by 6 day-old AB, ABstrg, *casper*, EK, TU and WIK larvae. ABstrg swam significantly further, and *casper*, EK, TU and WIK significantly less than AB. For the statistics refer to table 3. H) Mean distance swum in a 5-minute time interval by 1 month-old AB, ABstrg, *casper*, EK, TU and WIK juveniles. ABstrg swam significantly less, and EK, TU and WIK significantly further than AB and *casper*. For the statistics refer to table 3. I) Mean distance swum in a 5-minute time interval by 3 month-old AB, ABstrg, *casper*, EK, TU and WIK adults. ABstrg, EK, TU and WIK swam significantly less, and *casper* significantly further than AB. For the statistics refer to table 2.

We next looked at the development of fast swim events. These bursts of locomotion, which have also been interpreted as motor impulsivity, represent a behavioural parameter used in a zebrafish model of attention-deficit/hyperactivity disorder-like behaviour (ADHD) [13]. We compared fish of the AB, ABstrg, *casper*, EK, TU and WIK strains at 6 dpf, 1 month and 3 months of age. In this experiment, we first compared individual fish of each strain by plotting the total distance swum every 3 seconds during a 5-minute experiment. Representative traces of 3 fish from each stage show that there was a large amount of inter-individual variation at 6 dpf, 1 month and 3 months (Fig. 5A,B,C). Since data from individual fish were hard to analyse statistically, we counted the mean number of peaks of acceleration shown by single individuals of each strain during a 5-minute experiment. Fast swim events appeared to be highly variable at the three stages analysed. At 6 dpf, AB and ABstrg both showed 28 peaks of acceleration. In contrast to this, *casper*, EK, TU and WIK all showed significantly fewer peaks of acceleration (Fig. 5D). At 1 month of age, AB, *casper*, TU and WIK showed a similar number of peaks of acceleration. However, ABstrg and EK showed more peaks and thus an increased number of fast swim events (Fig. 5E). At 3 months, AB, *casper*, EK and WIK showed a similar number of peaks, whereas ABstrg and *casper* showed significantly fewer fast swim events (Fig. 5F). We also analysed the amount of acceleration within these peaks of locomotion. At 6pdf, ABstrg accelerated more, and *casper* and EK accelerated less than AB, TU and WIK (Fig. 5G). At 1 month, ABstrg accelerated less, and EK, TU and WIK more than AB and *casper* (Fig. 5H). Finally, at 3 months, *casper* accelerated less while ABstrg, EK, TU and WIK accelerated significantly faster than AB (Fig. 5I). In summary, these data show that the number of fast swim events is stable over developmental time with each strain showing between 22 and 30 peaks of acceleration at each stage of life. However, there were significant differences in both the number of peaks and the amount of acceleration in these peaks between strains. This suggests that the initiation of fast swim events was stable over time whereas fish steadily increased in their ability to produce bursts of high frequency swimming (for statistics, refer to table 3).

**Table 3:** Summary of the different *p* values following Student's t-test for the fast swim event experiment (number of peaks and speed in the peaks), described in Fig. 4D,E,F,G,H,I at 6 dpf, 1 month and 3 months.

						Fast	Swim	Events					
				Peaks						Speed			
		AB	ABstrg	*casper*	EK	TU	WIK	AB	ABstrg	*casper*	EK	TU	WIK
6 dpf	AB	-	NS	***	***	NS	***	-	***	***	***	***	***
6 dpf	ABstrg	NS	-	***	***	*	***	***	-	***	***	***	***
6 dpf	*casper*	***	***	-	NS	**	NS	***	***	-	*	***	***
6 dpf	EK	***	***	NS	-	***	NS	***	***	*	-	***	***
6 dpf	TU	NS	*	**	***	-	**	***	***	***	***	-	***
6 dpf	WIK	***	***	NS	NS	**	-	***	***	***	***	***	-
1month	AB	-	*	NS	NS	NS	NS	-	***	NS	***	***	***
1month	ABstrg	*	-	***	NS	NS	NS	***	-	***	***	***	***
1month	*casper*	NS	***	-	***	**	*	NS	***	-	***	***	***
1month	EK	NS	NS	***	-	NS	NS	***	***	***	-	***	NS
1month	TU	NS	NS	**	NS	-	NS	***	***	***		-	***
1month	WIK	NS	NS	*	NS	NS	-	***	***	***	NS	***	-
3month	AB	-	*	NS	NS	NS	NS	-	***	***	***	***	***
3month	ABstrg	*	-	NS	***	NS	**	***	-	***	***	***	***
3month	*casper*	NS	NS	-	NS	*	NS	***	***	-	***	***	***
3month	EK	NS	***	NS	-	***	NS	***	***	***	-	***	***
3month	TU	NS	NS	*	***	-	***	***	***	***	***	-	NS
3month	WIK	NS	**	NS	NS	***	-	***	***	***	***	NS	-

NS (non-significant), *p*>0.05;

* *p*<0.05,

** *p*<0.01,

*** *p*<0.001.

## Discussion

In this study we have compared the locomotor behaviour of six zebrafish strains as they mature from larval to adult stages. We chose to use AB as a reference to compare behaviour across strains. AB has been maintained in different laboratories for many years, its life-history is well characterised and it is freely available from Zebrafish International Resource Centre (ZIRC).

In order to improve handling of the large datasets generated in this experiment we developed novel software, “FastData Monitor”, that allows semi-automated analysis of several components of zebrafish locomotion. As zebrafish behavioural research becomes more popular the establishment of standardised protocols becomes crucial. Behavioural experiments are notoriously susceptible to large variation [22] and the use of software to automate behavioural analysis will help yield stable results across labs. With the behavioural setup described here we can test the same animals at 6 dpf (96 larvae), one month (40 juveniles) and three months (24 adults) in a medium throughput manner. Recent studies have shown that the zebrafish is an excellent choice for drug screens based on behaviour [23]–[25]. The protocols described here could form the basis of a standardised test battery to screen chemical compounds based on locomotion and/or the number of fast swim events. However, most zebrafish behavioural screens have been conducted at early stages precluding information about the possible effect of drugs in mature animals. The combination of standardised behavioural tests combined with automatic data sorting increases the potential for high-throughput drug screening at each stage of a zebrafish's life.

The locomotor parameters measured in this experiment (mean distance, speed and duration of swimming) were highly variable between strains and over time. For example, the rank order of distance swum between strains was not maintained. Several other studies have compared locomotion between different zebrafish lines. O'Malley and colleagues compared 7-day old *nacre* larvae to wild-type (strain not specified) and long-fin golden zebrafish larvae [26]. They observed a similar number of tail flicks following a touch to the head but did not quantify locomotor parameters such as swimming distance, speed and time in these animals [27]. De Esch and colleagues compared AB and Tupfel long fin (TL) wild-type fish. At 5 and 6 dpf, TL larvae showed a lower average distance swum than AB, whereas they swam further than AB at 7 days [28]. This study also reported that velocity (swimming speed) appears to be more affected by intrinsic and extrinsic factors (i.e. genetic and environmental influences) than total distance, a finding which is not in keeping with our 6 dpf data. Furthermore, we could not see any obvious correlation between parameters such as speed and distance of swimming and the number of peaks/speed of fast swim events. A number of environmental factors have also been shown to affect larval fish locomotion. These include the time of the day when behaviour is measured, the size of the arena in which animals were raised and exposure to conspecifics during development [29], [30]. Globally, the large amount of variability that we uncovered, both between strains and across time, strongly support the need to make baseline behavioural measurements before conducting analyses of mutant, morphant or transgenic zebrafish. The greater variability in fast swim events and the amount of time spent swimming compared to the speed and distance of locomotion suggest that both fast burst swimming and the duration of normal locomotion might either be controlled by different genes or be more susceptible to environmental influences.

The comparison between AB and ABstrg fish is particularly interesting. Since both fish stocks are derived from the same founder population, differences in their behaviour could be due to either environmental influences or random genetic drift. Previous work from our laboratory on an *fgfr1a* mutant fish showed that environmental factors, most likely acting during growth, can dramatically alter zebrafish aggression levels [31]. However, the effect of shipping the adult fish between Strasbourg and Gif-sur-Yvette is unlikely to have altered the outcome of our measurements, since acclimatisation to a new laboratory is sufficient to reduce behavioural differences in mouse [32]. The ABstrg animals tested here were born in Gif-sur-Yvette and grown in the same conditions as the other strains. It therefore seems likely that genetic drift between AB and ABstrg may account for the differences in behaviour that we observe.

Zebrafish siblings of the same strain show inter-individual variations in their locomotion levels despite being raised in standardised laboratory conditions. Genetic variation and developmental plasticity are fundamental properties of all living organisms. Although selection acts on phenotypic traits (including locomotion) its action is dependent on genetic heterogeneity. Recent research has identified large amounts of copy number variations (CNVs) between groups of AB, TU and WIK fish with TU showing the greatest polymorphism [33]. Furthermore, Howe and colleagues have identified around 7 million SNP differences between two homozygous zebrafish, representing a major source of genetic variation [34]. Coupled to environmental pressure in the lab, CNV variations could account for some of the differences that we see between AB and ABstrg. CNVs can directly influence the expression level of genes [33] with the potential to affect locomotion in multiple ways. Genetic heterogeneity might lead to alterations in the maturation of the central nervous system, musculature and appendages or changes to the overall body shape as discussed below. In order to directly compare levels of inter-strain polymorphism with changes to individual locomotor behaviour, the CNVs present in the families of fish used here would need to be characterised. If there are a similar number of CNVs, both between different strains and between individual members of each strain, then CNVs may not account for the alterations to behaviour which we observed.

In embryonic and larval zebrafish the recruitment of motoneurons, which form the interface between the central nervous system and musculature, follows the size principle [35]. At low swimming frequencies small motoneurons are active; as swimming speed increases, larger motoneurons are recruited expanding the pool of active cells [36]. Excitatory interneurons also modify the speed of swimming by setting the excitatory tone of locomotor networks [37]. The recruitment of motoneurons alters as a fish matures which, in concert with changes in descending input from supraspinal areas [35], [38] may contribute to the increase in speed and distance of swimming that we observe. The remarkable metamorphoses of body shape that zebrafish undergo between larval and adult stages can also explain some of these changes to locomotion [39]. During juvenile stages the larval median fin fold is replaced by paired pectoral and anal fins and the unpaired dorsal and caudal fins. Thus, both the distribution of propulsive surfaces and the drag forces that act upon the body are modified [40]. In both larval and adult fish the main swimming thrust is generated by trunk muscles, with momentum being transmitted to the water by the caudal fin [41]. The types of swimming exhibited by both larval and adult fish are also reflected by differential muscle use: red muscle fibres are recruited during slow swimming whereas fast swimming is driven by fast white muscle fibres [42]. Therefore, the steady increase in burst swimming over time might be caused by a combination of white muscle maturation [42], streamlining of the body and nervous system development. The size of a fish's body can also directly alter the biomechanics of locomotion. We were unable to measure the surface area of 6 dpf larvae due to their small size and the detection limit of our setup. However, at both 1 month and 3 months we were able measure fish using the ZebraLab software regardless of their pigmentation levels (for example, even though *casper* mutants are more transparent than AB fish their detectability was not affected; Fig. S2A–D). There was no correlation between surface area (an indirect measure of body size) and swimming speed at 1 month whereas at three months there was a weak negative correlation for AB, ABstrg and EK. These findings suggest that it is only at adult stages that a fish's body size impacts its locomotion.

The striking changes to body morphology and locomotion during larval and juvenile development raise questions about their behavioural significance. The ontogeny of locomotion could represent a by-product of nervous system and musculature development. Conversely, the different locomotion patterns of larval, juvenile and adult fish might constitute a specific adaptation at each life stage allowing the expression of age-specific behaviours. Many animal species display dramatic alterations to locomotion during development hinting at an important behavioural function. For example, *Xenopus laevis* switch from tail-based undulatory swimming as tadpoles to adult kick-based limb propulsion [43]. The locomotor performance of an animal can influence its fitness by altering prey capture, the ability to escape from predators and competition with siblings making it a target for selection [44]. In contrast to this, careful analysis of body morphology and the transition between viscous and inertial flow regimes in zebrafish suggests that locomotion is hampered by developmental constraints. For example, the change in zebrafish body morphology (and thus the influence of drag upon locomotion) during juvenile stages precedes the change between flow regimes [11]. Thus there appears to be a stage of larval growth in which the efficiency of locomotion is reduced due to the shape of the fish's body [11]. In agreement with this, the transition between different swimming patterns occurs rapidly during development, suggesting that the neural circuits which drive locomotion develop before a particular behaviour is expressed. Taken together, the pattern and timing of morphological and neurological development occurring before fish alter their locomotion is suggestive of developmental constraint acting upon this behaviour.

The large differences in behavioural ontogeny that we have documented, both between strains and over time, suggest that zebrafish behaviour is highly variable with modifications likely due to both genetic and environmental influences. Such fluctuations in behaviour complicate the standardisation and comparison of results between studies, a difficulty that is compounded by the absence of isogenic strains in zebrafish. Therefore, the results of this study indicate that a strain should be thoroughly characterised in each laboratory before behavioural experiments can be performed.

## Supporting Information

Figure S1 **Schematic representation of the 6 dpf, 1 month and 3 month behavioural setup.** At 6 dpf larvae were measured in 24-well plate in a ZebraBox. At one month the fish were individually placed in a small box (9.5 cm×6 cm×4.5 cm) inside a ViewPoint ZebraCube. At 3 months mature fish were placed in an AquaBox 3 and then positioned in a large home-made chamber that allowed 24 adult fish to be recorded at the same time.
(TIF)

Figure S2 **Photographs of** ***casper*** **and AB zebrafish in our behavioural setup.** Although *casper* mutant zebrafish are more transparent than AB wild-types, they were both readily detectable by the Zebralab programme. Comparison of adult AB and *casper* fish at 1 month (A,B) and 3 months (C,D).
(TIF)
