# Supplementary figures and images for: Inter-Individual and Inter-Strain Variations in Zebrafish Locomotor Ontogeny
Source: PLoS One. 2013 Aug 9;8(8):e70172. doi: 10.1371/journal.pone.0070172 (PMC3739779; doi:10.1371/journal.pone.0070172)

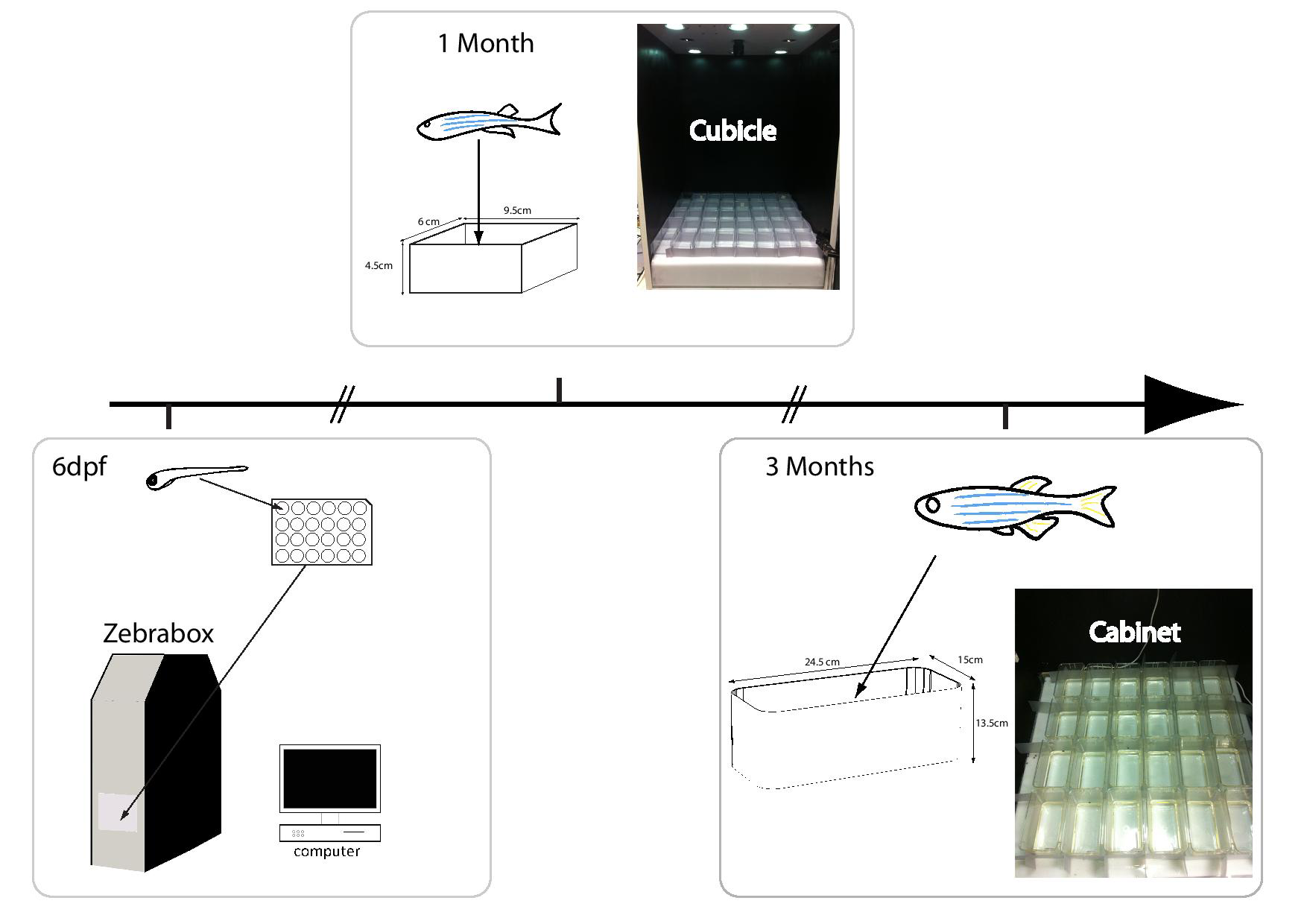

Supplement: Figure S1 — Schematic representation of the 6 dpf, 1 month and 3 month behavioural setup. At 6 dpf larvae were measured in 24-well plate in a ZebraBox. At one month the fish were individually placed in a small box (9.5 cm×6 cm×4.5 cm) inside a ViewPoint ZebraCube. At 3 months mature fish were placed in an AquaBox 3 and then positioned in a large home-made chamber that allowed 24 adult fish to be recorded at the same time. (TIF) [file pone.0070172.s001.tif]

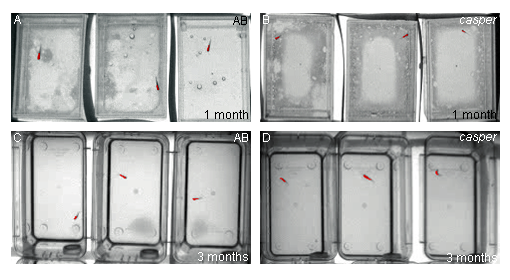

Supplement: Figure S2 — Photographs of casper and AB zebrafish in our behavioural setup. Although casper mutant zebrafish are more transparent than AB wild-types, they were both readily detectable by the Zebralab programme. Comparison of adult AB and casper fish at 1 month (A,B) and 3 months (C,D). (TIF) [file pone.0070172.s002.tif]
